# Using Targeted Resequencing for Identification of Candidate Genes and SNPs for a QTL Affecting the pH Value of Chicken Meat

**DOI:** 10.1534/g3.115.020552

**Published:** 2015-08-14

**Authors:** Xidan Li, Xiaodong Liu, Javad Nadaf, Elisabeth Le Bihan-Duval, Cécile Berri, Ian Dunn, Richard Talbot, Dirk-Jan De Koning

**Affiliations:** * Swedish University of Agricultural Sciences, SE-750 07 Uppsala, Sweden,; † Department of Ecology and Genetics, Plant Ecology and Evolution, Uppsala University, SE-752 36 Uppsala, Sweden,; ‡ INRA, UR83 Recherches Avicoles, Nouzilly, 37380, France,; § Roslin Institute and R(D)SVS, University of Edinburgh, Midlothian, EH25 9RG, United Kingdom

**Keywords:** chicken muscle, pH values, QTL, next-generation sequencing, SNPs, nonsynonymous SNPs

## Abstract

Using targeted genetical genomics, a quantitative trait locus (QTL) affecting the initial postmortem pH value of chicken breast muscle (*Pectoralis major*) on chromosome 1 (GGA1) recently was fine-mapped. Thirteen genes were present in the QTL region of approximately 1 Mb. In this study, 10 birds that were inferred to be homozygous for either the high (QQ) or low (qq) QTL allele were selected for resequencing. After enrichment for 1 Mb around the QTL region, >500 × coverage for the QTL region in each of the 10 birds was obtained. In total 5056 single-nucleotide polymorphisms (SNPs) were identified for which the genotypes were consistent with one of the QTL genotypes. We used custom tools to identify putative causal mutations in the mapped QTL region from these SNPs. Four nonsynonymous SNPs differentiating the two QTL genotype groups were identified within four local genes (PRDX4, EIF2S3, PCYT1B, and E1BTD2). Although these are likely candidate SNPs to explain the QTL effect, 54 additional consensus SNPs were detected within gene-related regions (untranslated regions, splicing sites CpG island, and promoter regions) for the QQ birds and 71 for the qq birds. These could also play a role explaining the observed QTL effect. The results provide an important step for prioritizing among a large amount of candidate mutations and significantly contribute to the understanding of the genetic mechanisms affecting the initial postmortem pH value of chicken muscle.

Meat quality traits such as color, water-holding capacity, and texture are important in poultry and are determined by both genetic and environmental factors. The postmortem decrease in pH is a key factor affecting these meat quality traits (Berri *et al.* 2007). Meat with a low pH value tends to be pale in color with low water-holding capacity, which has a critical impact on quality of further processed products (Dransfield 1999; Alnahhas *et al.* 2014). Previous studies have shown that ultimate pH of meat is highly dependent on the amount of glycogen in the muscle (Le Bihan-Duval *et al.* 2008). This glycogen rapidly is depleted in the muscle while the birds are exposed to stress (Ngoka and Froning 1982) and exhibit high physical activity (Debut *et al.*, 2005) before slaughter. Therefore, preslaughter stress may be associated with variation in the initial rate of decrease in pH (Chabault *et al.*, 2012). At the genetic level, a recent fine mapping study has characterized a quantitative trait locus (QTL) affecting muscle pH measured 15 min postmortem (pH 15) using an F_2_ intercross between High-Growth (HG) and Low-Growth (LG) chicken lines (Nadaf *et al.* 2007, 2014). The HG and LG lines are bidirectionally selected on body weight at 9 wk of age. The HG lines show lower pH 15 values, and the LG lines show greater pH 15 values as a correlated response to selection for growth. Identifying the causal genetic component underlying this QTL region would advance our understanding of the genetic architecture of poultry muscle metabolism and potentially poultry stress behavior.

In this study, 10 birds that were homozygous at the targeted QTL (QQ *vs.* qq) were selected for resequencing, where QQ corresponds to alleles from the HG lines and qq corresponds to alleles from the LG lines (Nadaf *et al.* 2014). Based on the bioinformatics analysis of the mapped QTL region and single-nucleotide polymorphisms (SNPs) detected by next-generation sequencing (NGS) data, we report a number of candidate genes and SNPs.

## Materials and Methods

### Study samples

In this study, we selected 10 birds from the F2 with inferred QTL genotypes of QQ (five birds, homozygous for HG line alleles) or qq (five birds, homozygous for LG line alleles). The selected individuals are a subset of the birds that were used for the targeted genetical genomics study by Nadaf *et al.* (2014) selected for the lowest relatedness within genotype.

### Remapping the QTL region in the Galgal4.0 genome

**Figure 1:**
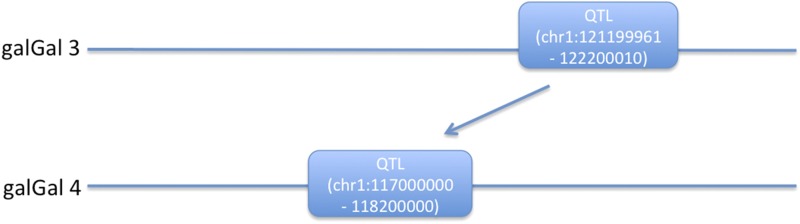
Remapping the quantitative trait locus (QTL) region from the Galgal3.0 genome to the Galgal4.0 genome.

The previously fine-mapped QTL region, which spanned less than 1 Mbps on chromosome 1, was resequenced based on the reference genome Galgal3.0. However, with the recently assembled reference genome Galgal4.0, the updated gene information had some differences compared with Galgal3.0 for our target region. Therefore, we remapped the QTL region onto the genome assembly Galgal4.0, where sequence of the fine-mapped QTL region on genome assembly Galgal3.0 was blasted against the genome sequence Galgal4.0 (Zhang *et al.* 2000). As a result, the new QTL region of around 1.2 Mbps has been mapped to chr1: 117000000−118200000 (on galGal4.0; Figure 1, supporting information, Table S1).

### NGS analysis

Target enrichment analyses and next generation sequencing were performed by Edinburgh Genomics (https://genomics.ed.ac.uk/). To obtain high coverage in the QTL region, Agilent SureSelect (Agilent, Santa Clara, CA) target enrichment system was used to perform DNA sequence enrichment using probes from the QTL region according to genome assembly Galgal3.0. SureSelect technology is designed to isolate a subset (up to 24 Mb) of a genome region or regions for high throughput sequencing. Sequencing was performed with the use of Illumina paired-end libraries with 150 bps for each pair of reads and an average insert size of 300 bps.

The Illumina Genome Analyzer Analysis Pipeline was used to produce paired sequence files containing reads for each sample in Illumina FASTQ format. All sequences have been deposited in the Sequence Read Archive (SRA; http://www.ncbi.nlm.nih.gov/sra) under accession number SRP051545. The sequences alignments were done using bowtie (Langmead *et al.* 2009) based on the remapped QTL region on the genome Galgal4.0, where about 500× coverage for each of 10 homozygote genotypes was achieved. Furthermore, samtools (Li *et al.* 2009) was used to perform SNP-calling, and the resulting VCF files were used for further candidate gene analysis (workflow provided in File S1 and sequencing details of the target QTL region provided in Table S1).

### Detecting potential causative genes and SNPs

**Figure 2:**
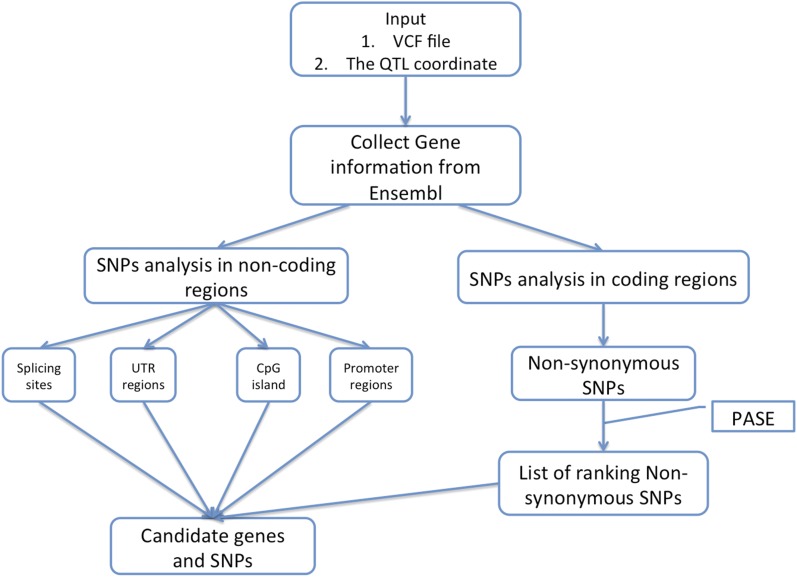
Schematic overview of the workflow to identify the candidate genes and single-nucleotide polymorphisms (SNPs).

The QTL region affecting the pH 15 value of chicken meat has been fine mapped previously (Nadaf *et al.* 2014). Although QTL analysis has been shown to be useful to detect the genetic variations related to the common complex trait and genotype, the ability to detect the causative functional genes and SNPs is very limited. In this study, we developed a new strategy to identify the functional SNPs in known genes in a defined QTL region, which could prioritize the most promising SNPs among a large pool of candidates (workflow and subroutines presented in File S2 and File S3). From the VCF files, the gene information such as gene’s transcript id, the coordinate of RNA splicing sites, and untranslated regions (UTR) in the remapped QTL region were retrieved from Ensembl (Flicek *et al.* 2013). Next, SNPs located within the noncoding regions such as RNA splicing sites, and UTR regions were identified and recorded as potential factors affecting gene expression. SNPs in the coding regions that were nonsynonymous mutations were evaluated and ranked according to their effect on the protein function by PASE (Li *et al.* 2013; https://github.com/xidan21/PASE) (Figure 2). Finally, the identified SNPs from each sample were categorized according to their genotypes. The SNPs that are present in, almost, every sample of one QTL genotype group (*e.g.*, QQ) and absent in the other QTL genotype group are called “consensus SNPs.” These consensus SNPs were chosen as candidate SNPs, and the corresponding genes were prioritized for future analyses.

### Data availability

Sequence data from this article have been deposited with the Sequence Read Archive (SRA; http://www.ncbi.nlm.nih.gov/sra) under accession no. SRP051545. File S1 contains the script package to perform sequence alignment and SNP calling. File S2 contains the scripts to annotate and prioritize the SNPs while File S3 contains examples of output files and further example scripts. The PASE software is accessible via https://github.com/xidan21/PASE.

## Results and Discussion

In this study we identified 2593 SNPs that were concordant with in the QQ QTL genotype and 2513 SNPs that were concordant with the qq QTL genotype (Table S2 and Table S3). Of these, 129 SNPs could be linked to candidate genes (Table 1, Table S4, and Table S5). Among these, four nonsynonymous single-nucleotide polymorphisms (nsSNPs) were identified within four genes (Table 2). One consensus nsSNP located in the gene *PRDX4* was detected in four of five QQ birds. Previous studies show that the pH 15 value of chicken meat is associated with straightening up and wing flapping at the slaughter shackle line (Berri *et al.* 2005; Nadaf *et al.* 2007). These activities at the slaughter line could directly result in fatigue of the muscle by the production of oxidants. Therefore, oxidative stress has been suggested to contribute to the pH 15 value of chicken meat (Nadaf *et al.* 2014). Interestingly, *PRDX4*, as one member of the antioxidant enzyme family, plays a crucial role in the process of protecting muscle cells against oxidative stress (Novelli *et al.* 1991; Leyens *et al.* 2003). Our previous study has shown that the expression level of *PRDX4* is significantly different between the QQ and qq genotype groups (Nadaf *et al.* 2014). Subsequently, we used our in-house developed software PASE to evaluate the effect of the nsSNP in *PRDX4*. As shown in Table 2, the mutation (T179P) caused by the nsSNP in *PRDX4* has a high conservation score (0.79) and a low physicochemical properties changes score (0.22). The nsSNP in *PRDX4* with a high conservation score is expected to contribute to the considerable difference in *PRDX4* expression between the QTL genotypes (Nadaf *et al.* 2014). Therefore, *PRDX4* remains a strong candidate gene for this QTL. The three other nsSNPs were detected within three genes: *EIF2S3*, *PCYT1B*, and *E1BTD2* (Table 2). *EIF2S3* has been described to contribute to the early stage of protein synthesis by interacting with guanosine-5′-triphosphate and initiator transfer RNA to form a ternary complex and binding to a 40S ribosomal subunit (Entrez Gene: *EIF2S3*). *PCYT1B* is an enzyme and involved in the regulation of phosphatidylcholine biosynthesis. Several alternatively spliced transcript variants encoding different isoforms have been found for this gene (Wang *et al.* 2005). Another gene, *E1BTD2*, is uncharacterized. By using BLAST, the predicted function of *E1BTD2* was “organic solute transporter subunit alpha-like,” which is involved in the activity of transportation from the endoplasmic reticulum to the plasma membrane (Dawson *et al.* 2005). By using PASE, the amino acid changes caused by these nsSNPs also show high conservation scores, which indicates these nsSNPs can affect the expression or function of the corresponding protein (Table 2). However, these genes (*EIF2S3*, *PCYT1B*, and *E1BTD2*) were not differentially expressed (FDR >0.20) in the previous study (Nadaf *et al.* 2014). In metabolic studies, it has been shown that substantial changes in metabolites can be caused by subtle differential expression of the underlying genes (Brown 1992; Mootha *et al.* 2003). In summary, these genes can all be linked to the energy metabolism and thus remain positional candidate genes for the QTL.

**Table 1:** Number of consensus SNPs in gene-related regions including UTR, splicing site, CpG island, and promoter regions differentiating the QQ and qq genotypes for a QTL affecting pH after slaughter on chicken chromosome 1

Genotypes	nsSnps	UTR	Splicing Sites	CpG Islands	Promoter Regions	Total
QQ	3	28	1	3	23	57
qq	1	25	3	5	38	72
Total	4	53	4	8	61	129

Details of corresponding genes and SNP locations are in Table S4 and Table S5. SNP, single-nucleotide polymorphism; UTR, untranslated region, QTL, quantitative trait locus.

**Table 2:** Consensus nonsynonymous SNPs differentiating the QQ and qq genotypes for a QTL affecting pH after slaughter on chicken chromosome 1

Gene ID	Genotype	Nonsynonymous SNPs			PASE Scores*a*	
		Coordinate	Codon (Ref/Alt)	Amino Acid Substitution	Physicochemical Properties Scores	Sequence Conservation Scores
PRDX4	QQ	117796698	**A**CT/**C**CT (4/5)*b*	T179P	0.22	0.79
EIF2S3	QQ	117669996	**G**GT/**A**GT	G335S	0.26	0.83
E1BTD2	QQ	117608741	**T**TT/**A**TT	F129I	0.19	0.83
PCYT1B	qq	117503317	G**C**A/G**G**A	A44G	0.22	0.86

SNP, single-nucleotide polymorphism; QTL, quantitative trait locus.

a PASE score comprises physicochemical properties scores and sequence conservation scores. Each of score ranges from 0 and 1, where 0 is neutral, and the greater ratio indicates the stronger effect on the hosting protein.

b The consensus nsSNP located in the gene PRDX4 has been detected in four of a total of five in the QQ genotype samples.

Moreover, 54 consensus SNPs were detected within gene-related regions (UTR regions, splicing sites, CpG islands, and promoter regions) for the QQ birds and 71 for the qq birds (Table 1, Table S4, and Table S5). Because variations in regulatory elements are expected to contribute to variation in complex traits, these consensus SNPs could affect the expression of target genes and contribute to the observed QTL effect. For example, in the QQ group, one consensus SNP was identified at a canonical splicing site of *ACOT9*, which could result in inhibition of splicing and have a decisive impact on its function. Other good examples are *APOO* and *KLHL15*. For *APOO*, five consensus SNPs in the CpG islands were identified between two groups (three SNPs in QQ and two SNPs in qq). For *KLHL15*, in the QQ group, five consensus SNPs in the UTR region were identified, whereas in the qq group, five consensus SNPs in UTR region and six consensus SNPs in the promoter region were identified (Table S4 and Table S5). These consensus SNPs in regulatory regions of *APOO* and KLHL15 could result in an incorrect transcription that can cause in the change or absence of the corresponding proteins. In addition, *ACOT9*, *APOO*, and *KLHL15* were differentially expressed in both the microarray teal = >real time polymerase chain reaction analysis between QQ and qq groups in the previous study (Nadaf *et al.* 2014). Thus, *ACOT9*, *APOO*, and *KLHL15* also are strong candidate genes for the QTL effect.

In the current study, we used target enrichment to get high coverage of our QTL region in the sequence data. This strategy has been successful in terms of creating high average coverage but there are also some drawbacks: Because the target enrichment was based on 1 Mb on genome build Galgal3.0, the remapped region of 1.2 Mb on genome build Galgal4.0 means that we cannot have continuous sequence coverage of the QTL region. Furthermore, the target enrichment process ignores repetitive DNA sequences in the target region, causing additional problems for having continuous coverage. These factors mean that the current resequencing may have missed polymorphisms in the target region. Given the current prices for sequencing, we would recommend to do a full genome sequence of these target birds rather than only the QTL region, even though repetitive DNA would still be a challenge.

In conclusion, the results confirm that *PRDX4*, *ACOT9*, *APOO*, and *KLHL15* are strong candidates for the QTL affecting the pH 15 value of chicken meat. In addition, *EIF2S3*, *PCYT1B*, and *E1BTD2* with consensus nsSNPs also are potential candidate genes for the observed QTL effect although the role of metabolism mechanism underlying chicken meat pH values still is not completely clear. The list of plausible candidate genes and mutations from the present study will facilitate further verification and experimental evaluation. With the current study we have fully exploited the potential for fine mapping in the divergent F2 cross. To differentiate among the current list of candidate gens and SNPs we will need additional experiments. For instance, the candidate SNPs could be tested on a broader panel of birds from different breeds to evaluate their putative effect. Thus, an improved understanding of the genetic basis of variation in pH values will assist selection of breeding birds.

## 
